# Epigenetic Reprogramming During Somatic Cell Nuclear Transfer: Recent Progress and Future Directions

**DOI:** 10.3389/fgene.2020.00205

**Published:** 2020-03-18

**Authors:** Xiangyu Wang, Jiadan Qu, Jie Li, Hongbin He, Zhonghua Liu, Yanjun Huan

**Affiliations:** ^1^College of Veterinary Medicine, Qingdao Agricultural University, Qingdao, China; ^2^Department of Cadre Health Care, Qingdao Municipal Hospital, Qingdao, China; ^3^College of Life Sciences, Shandong Normal University, Jinan, China; ^4^College of Life Sciences, Northeast Agricultural University, Harbin, China

**Keywords:** somatic cell nuclear transfer, cloning efficiency, nuclear reprogramming, epigenetic modification, long non-coding RNA

## Abstract

Somatic cell nuclear transfer (SCNT) has broad applications but is limited by low cloning efficiency. In this review, we mainly focus on SCNT-mediated epigenetic reprogramming in livestock and also describe mice data for reference. This review presents the factors contributing to low cloning efficiency, demonstrates that incomplete epigenetic reprogramming leads to the low developmental potential of cloned embryos, and further describes the regulation of epigenetic reprogramming by long non-coding RNAs, which is a new research perspective in the field of SCNT-mediated epigenetic reprogramming. In conclusion, this review provides new insights into the epigenetic regulatory mechanism during SCNT-mediated nuclear reprogramming, which could have great implications for improving cloning efficiency.

## Introduction

**FIGURE 1:**
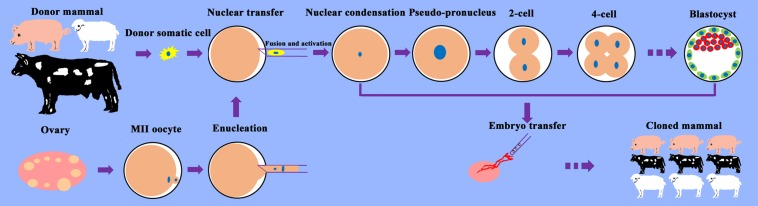
Schematic illustration of the SCNT process. Somatic cells from the desired donor mammal are cultured for SCNT. Oocytes are recovered from the ovaries obtained from the slaughterhouse and allowed to mature into metaphase (M)II oocytes. MII oocytes are enucleated, and donor somatic cells are transferred into the perivitelline space of oocytes. After the fusion and activation of cell–cytoplast complexes, the reconstructed cloned embryos begin to develop, undergoing nuclear condensation and nuclear swelling, followed by pseudo-pronucleus, 2-cell, 4-cell, etc. stages and form blastocysts *in vitro*. Cloned embryos are transferred into surrogates and develop into cloned mammals.

Somatic cell nuclear transfer (SCNT) is an assisted reproduction technology for the generation of cloned mammals that involves the culture of donor somatic cells and oocytes, transplantation of donor cell nuclei into enucleated oocytes, activation of reconstructed embryos, and transfer of cloned embryos into surrogates (Figure 1). SCNT enables the reprogramming of terminally differentiated cells into totipotent cells, which has revolutionized our understanding of cell fate determination and development, and has significant value for theoretical research and production applications.

## SCNT in Mammals

The first SCNT mammal was a sheep known as Dolly that was born in 1997 (Wilmut et al., 1997), and since then, SCNT has entered a new era (Table 1). A series of cloned mammals, including cow, mouse, goat, pig, and cat, have been produced with this technology (Cibelli et al., 1998; Wakayama et al., 1998; Baguisi et al., 1999; Polejaeva et al., 2000; Chesne et al., 2002; Shin et al., 2002; Galli et al., 2003; Woods et al., 2003; Zhou et al., 2003; Lee et al., 2005; Li et al., 2006; Berg et al., 2007; Shi et al., 2007; Wani et al., 2010). In 2018, the first non-human primate species, the macaque monkey, was successfully cloned by SCNT, further attracting worldwide attention on SCNT technology (Liu et al., 2018; Matoba and Zhang, 2018). Using this technology, a large number of mammals have been successfully produced, showing many potential applications (Campbell et al., 2007; Ogura et al., 2013; Telugu et al., 2017). In agriculture, SCNT can rescue endangered species, protect the genetic resources of commercially important species, and accelerate the propagation of breeding livestock, including pigs, cows, and sheep (Gomez et al., 2009; Keefer, 2015). In combination with genome-modification technologies such as the recently developed clustered regularly interspaced short palindromic repeats (CRISPR)/CRISPR-associated protein 9-mediated genome editing, SCNT can rapidly produce cloned mammals with desirable traits including rapid growth, disease resistance, and good meat quality, thereby cultivating novel varieties, and shortening breeding cycle (Galli et al., 2012; Wells and Prather, 2017; Lee et al., 2020). In biomedicine, SCNT can create a mammary gland bioreactor to produce therapeutic proteins, establish animal models to investigate the pathogenesis of human diseases, and produce genetically modified xenograft organs for patient transplantation (Lotti et al., 2017; Niu et al., 2017; Telugu et al., 2017). SCNT can also generate blastocyst-derived stem cells, namely, nuclear transfer embryonic stem cells (ntESCs), especially human ntESCs, which are isogenic to the donor and do not cause immune rejection when transplanted, thus providing an important tool for organ regeneration (Tachibana et al., 2013). In basic research, SCNT has been used to investigate interactions between the nucleus and cytoplasm, which has enhanced our understanding of the mechanisms of cell fate determination (Long et al., 2014). Moreover, SCNT has promoted the generation and development of induced pluripotent stem cells (iPSCs), which have similar therapeutic applications to ntESCs (Takahashi and Yamanaka, 2006).

**TABLE 1:** Mammals first cloned by different SCNT-based procedures.

Mammal	Special procedures		
	Donor cells	Oocytes	Cloned embryos
Sheep	Synchronized adult mammary epithelial cells	Superovulated MII oocytes	General SCNT
Cow	Transgenic fetal fibroblast cells	Oocyte maturation *in vitro*	General SCNT
Mouse	Adult cumulus cells without *in vitro* culture	Superovulated MII oocytes	Donor cells injected into enucleated oocytes
Goat	Synchronized transgenic fetal fibroblast cells	Superovulated MII and TII oocytes	General SCNT
Pig	Synchronized adult granulosa cells	Superovulated MII oocytes and zygotes	Double nuclear transfer
Cat; ferret	Adult cumulus cells	Oocyte maturation *in vitro*	General SCNT
Rabbit	Adult transgenic cumulus cells	Superovulated MII oocytes	General SCNT
Mule	Fetal fibroblast cells	Oocyte collection *in vivo*	General SCNT
Horse	Adult fibroblast cells	Oocyte maturation *in vitro*	Zona-free manipulation
Rat	Synchronized fetal fibroblast cells	Oocyte maturation *in vivo*; blocking oocyte activation	One-step SCNT
Dog	Adult fibroblast cells	Oocyte maturation *in vivo*	General SCNT
Buffalo	Synchronized fetal fibroblast and adult granulosa cells	Oocyte maturation *in vitro*	General SCNT
Red deer	Antlerogenic periosteum, putative bone and fat cells	Oocyte maturation *in vitro*	General SCNT
Camel	Adult cumulus cells	Oocyte maturation *in vivo*	General SCNT
Macaque monkey	Fetal fibroblast cells	Oocyte maturation *in vivo*	HVJ-E-mediated fusion; embryos with kdm4d injection and TSA treatment

*General SCNT refers to the fact that donor cell is transferred into the perivitelline space of enucleated MII oocyte and the cell–cytoplast complexes are fused by electrical stimulation and activated by electrical or chemical induction.*

## Low Cloning Efficiency Limits the Applicability of SCNT

Although SCNT has been successfully used to clone many species of mammals with significant improvements in cloning efficiency in more than 20 years since the birth of Dolly, the proportion of cloned embryos that develop to full term remains very low, greatly limiting the application of SCNT technology (Czernik et al., 2019). To improve the birth of cloned mammals and cloning efficiency, researchers have investigated the effects of donor cell type, oocyte maturation stage, embryo activation method, etc. on the developmental competence of cloned embryos (Table 1), and to some extent, cloning efficiency has been shown to increase through optimizing these parameters (Blelloch et al., 2006; Campbell et al., 2007; Kurome et al., 2013). However, cloning efficiency remains low, the abortion of cloned fetus frequently occurs, and the rate of abnormality or mortality is high. Moreover, developmental defects still occur in cloned mammals even after birth (Campbell et al., 2007; Loi et al., 2016). These phenomena demonstrate that optimizing these technology parameters of SCNT cannot make significant improvements in cloning efficiency and only clarifying the theoretical molecular mechanism underlying SCNT could understand the cause of the poor and abnormal development of cloned embryos. However, SCNT-mediated nuclear reprogramming is still poorly understood, and the key factors determining the developmental potential of cloned embryos remain unclear (Matoba and Zhang, 2018). Therefore, fully and clearly revealing the molecular mechanism underlying SCNT-mediated nuclear reprogramming is needed to enhance the development of cloned embryos.

## Incomplete Epigenetic Reprogramming Underlies Low Cloning Efficiency

**FIGURE 2:**
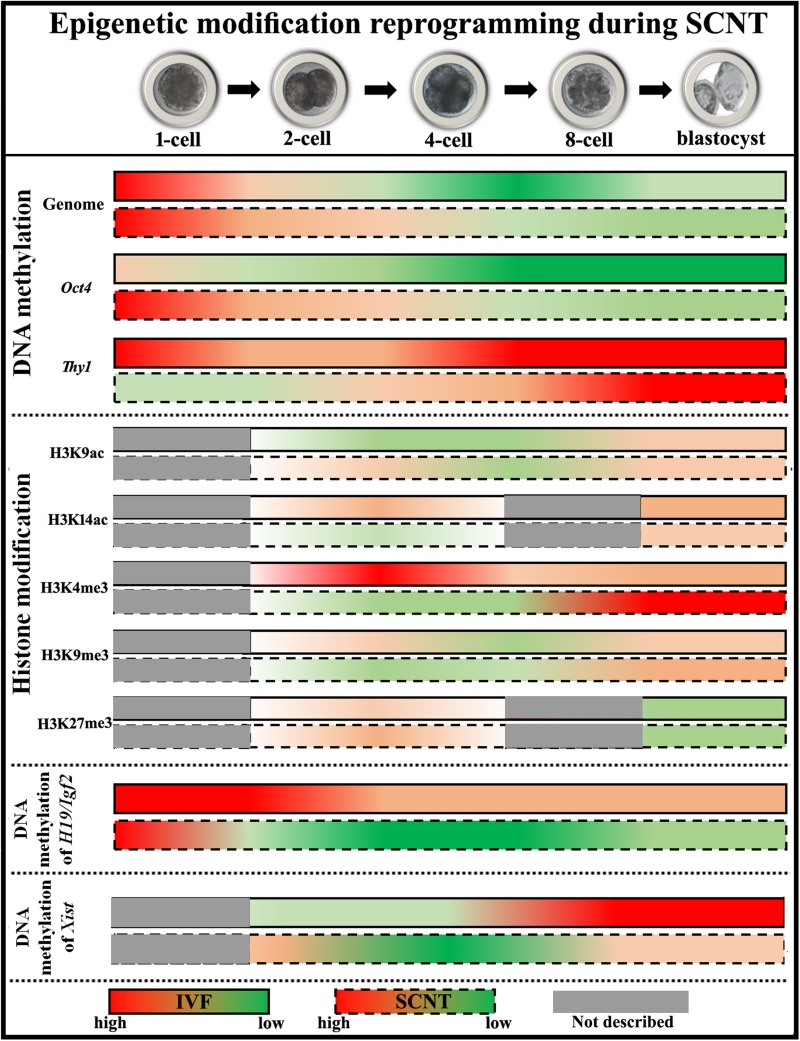
Diagram of epigenetic modification changes during SCNT-mediated nuclear reprogramming. The data of pig embryos are adopted to describe epigenetic modification reprogramming. For DNA methylation, cloned embryos demonstrate delayed DNA demethylation and incomplete DNA remethylation of genome (the data shown here represent DNA methylation status at centromeric repeats, which partly reflects genome DNA status), high DNA methylation status of pluripotency-related gene *Oct4*, and low DNA methylation levels of tissue-specific gene *Thy1*, respectively. For histone modifications, low levels of histone acetylation (H3K9ac at the ZGA stage and H3K14ac at the blastocyst stage) and H3K4me3, and high levels of histone methylation (H3K9me3 after ZGA and H3K27me3 at the 2-cell stage) are observed in cloned embryos. For genomic imprinting, DNA methylation of *H19/Igf2* is not maintained during SCNT. For XCI, DNA methylation of *Xist* is not fully established in female cloned embryos.

During development, totipotent embryos differentiate into pluripotent stem cells and subsequently into differentiated cells. Cell fate determination is largely achieved by activating some genes while suppressing other genes through epigenetic modification such as DNA methylation, histone modification, genomic imprinting, and X chromosome inactivation (XCI) (Reik et al., 2003). These heritable changes in gene expression without alterations in genomic DNA sequences occur during the progression from fertilized oocyte to differentiated embryo and also play a key role in embryo development following SCNT (Niemann, 2016). It is thought that the low cloning efficiency, abnormal embryo phenotype, and low viability of animals generated by SCNT are due to incomplete reprogramming of donor nuclei (Yang et al., 2007). Epigenetic changes during the SCNT process are discussed in greater detail below (Figure 2).

DNA methylation occurs at cytosine residues in the CpG dinucleotide and is generally associated with transcriptional silencing (Schubeler, 2015). In the life cycle, the genome undergoes DNA methylation maintenance, DNA demethylation, and DNA remethylation, which allows organisms to activate or silence specific genes according to the requirements of organism growth and development (Li and Zhang, 2014). DNA methyltransferases (Dnmts) such as Dnmt1 and Dnmt3 (Dnmt3a, Dnmt3b, and Dnmt3l) are responsible for DNA methylation maintenance and *de novo* DNA methylation (Chen and Zhang, 2019). DNA demethylation occurs through the oxidation-base excision repair pathway. Oxidative DNA demethylation enzymes include ten–eleven translocation (Tet)1, Tet2, Tet3, activation-induced cytidine deaminase, and DNA glycosylases (Ito et al., 2010; Iqbal et al., 2011; Shen et al., 2013). Other pathways also contribute to active DNA demethylation during early embryonic development (Wang et al., 2014). Dnmt1 maintains methyl marks on genomic DNA and ensures that the DNA methylation pattern of offspring cells is identical to that of parental cells (Lyko, 2018). After fertilization, the genome demonstrates a combination of active and passive DNA methylation, and the paternal genomic DNA is actively demethylated while maternal genomic DNA is passively demethylated (Guo et al., 2014). When fertilized embryos develop to the blastocyst or subsequent implantation stage, genomic DNA is remethylated (Reik et al., 2001; Yang et al., 2007). In cloned embryos, the genomic DNA of donor somatic cells is highly methylated and DNA methylation reprogramming (especially DNA demethylation) is necessary for development to proceed normally. The genome also undergoes de-/remethylation during SCNT, but this is delayed and incomplete compared with normal embryos (Bourc’his et al., 2001; Dean et al., 2001; Yang et al., 2007). Tissue-specific and pluripotency-related genes in cloned embryos show low and high DNA methylation levels, respectively (Figure 2, DNA methylation) (Ng and Gurdon, 2005; Kremenskoy et al., 2006; Yamazaki et al., 2006; Huan et al., 2014, 2015a). Following zygotic genome activation (ZGA), the erroneous reconstitution of DNA methylation pattern caused by aberrant expression of genes related to DNA methylation reprogramming and, consequently, of key genes required for the normal development of cloned embryos results in low cloning efficiency and abnormalities and death in cloned animals (Bourc’his et al., 2001; Bortvin et al., 2003; Chung et al., 2003; Kiefer et al., 2016; Gao et al., 2018). Thus, a DNA methylation pattern similar to that in normal fertilized embryos is necessary for the successful development of SCNT embryos.

Chromatin structure and histone modification are key factors that regulate gene expression (Sproul et al., 2005; Yi and Kim, 2018). The basic structural unit of chromatin is the nucleosome, a histone octamer consisting of two copies each of H2A, H2B, H3, and H4 wrapped by 146 bp of DNA and H1 as a linker (Kobayashi and Kurumizaka, 2019). Gene expression depends on chromatin accessibility, which is controlled by chromatin remodeling factors and through covalent modification (e.g., acetylation, methylation, and phosphorylation) of amino acids in the histone tail (Qin et al., 2016; Kobayashi and Kurumizaka, 2019). Chromatin accessibility during the SCNT-mediated epigenetic reprogramming has not been extensively investigated as it requires a large number of embryos. Recently, progress is being made in mice owing to technologic advances such as low-input DNase I hypersensitive site (DHS) sequencing and transposase-accessible chromatin sequencing (Wu et al., 2016; Djekidel et al., 2018). DHSs, which are positively correlated with gene expression, are present in donor somatic cells and are reprogrammed in cloned embryos. However, specific DHSs of donor somatic cells fail to be reprogrammed to those of embryos, which prevents the binding of chromatin remodeling factors to regulate gene expression in cloned embryos (Djekidel et al., 2018).

Histone acetylation is regulated by histone acetyltransferase (Hat) and histone deacetylase (Hdac). Hat opens up chromatin, which allows transcription factor binding and leads to activation of gene transcription, whereas Hdac promotes gene inactivation (Sun et al., 2003). After fertilization, histone acetylation such as histone H3 acetylation occurs and allows the appropriate expression of genes related to early embryonic development (Rybouchkin et al., 2006; Ziegler-Birling et al., 2016). During SCNT, histone acetylation marks decrease and gradually disappear, for instance, Lys9 acetylation of H3 (H3K9ac) at the ZGA stage and H3K14ac at the blastocyst stage (Rybouchkin et al., 2006; Wee et al., 2006; Liu et al., 2012; Zhai et al., 2018). Histone methylation mainly occurs on lysine and arginine, and involves three methylation patterns including monomethylation, dimethylation, and trimethylation (Izzo and Schneider, 2010). Trimethyl of H3 Lys4 (H3K4me3) and H3K27me3 are the most typical modifications. H3K4me3 is regulated by the Trithorax group (TrxG) complex and is associated with gene activation, while H3K27me3 is mediated by the Polycomb group (PcG) proteins and leads to gene silencing (Liu X. et al., 2016). During SCNT-mediated nuclear reprogramming, H3K4me3 level decreases and H3K27me3 level increases (Cao et al., 2015; Xie et al., 2016; Zhai et al., 2018; Zhou et al., 2019). Another modification, H3K9me3, is catalyzed by suppressor of variegation 39H1/2 (Suv39H1/2) and removed by lysine demethylase (Kdm)4 (Kdm4a, Kdm4b, Kdm4d, and Kdm4e), and can alter chromatin conformation to inhibit gene expression (Ninova et al., 2019). H3K9me3 can be removed in donor somatic cells, but incomplete H3K9me3 demethylation in cloned embryos inhibits their development. Studies have shown that H3K9me3 is enriched in the promoters of genes against SCNT-mediated nuclear reprogramming, suggesting that incomplete H3K9me3 demethylation is an inhibitor of the development of cloned embryos (Matoba et al., 2014; Liu W. et al., 2016; Zhai et al., 2018). These disrupted histone modifications finally affect chromatin accessibility, lead to the disordered expression of genes required for the normal development of cloned embryos, and result in low cloning efficiency (Figure 2, Histone modification) (Liu et al., 2012; Xie et al., 2016; Zhai et al., 2018). Several histone variants also exhibit abnormalities such as the delayed change of H1foo (oocyte-specific H1) to somatic H1s, macroH2A expression before the endogenous activation, and the existing replacement of donor cell H3 carrying repressive modification by maternal H3.3 in cloned embryos, which contributes to incomplete SCNT-mediated nuclear reprogramming (Gao et al., 2004; Chang et al., 2010; Wen et al., 2014). Therefore, histone modification is a critical determinant in the development of cloned embryos.

Genomic imprinting, an epigenetically regulated phenomenon that shows monoallelic parent-specific gene expression, is controlled by the differentially methylated region (DMR) or specific histone modifications. The DMR is protected by DNA-binding complexes composed of Dnmt1, zinc finger protein 57, and tripartite motif-containing 28, and the H3K27me3 mark (Barlow and Bartolomei, 2014; Inoue et al., 2017a). In general, paternal and maternal gene imprinting promotes and inhibits, respectively, offspring growth and development (Barlow and Bartolomei, 2014). Therefore, parental imprinted genes compete with or complement each other, and the balance between the expression of paternal and maternal imprinted genes is important for normal developmental progression. H19/insulin-like growth factor (Igf)2 is a typical genomic imprinting locus with the DMR methylated on the paternal allele, on which H19 silencing stimulates IGF2 activity and cell growth. In contrast, H19 on the maternal allele is an inhibitory factor that has a cis-silencing effect on Igf2 expression. Inhibiting Igf2 expression leads to fetal growth retardation, whereas Igf2 overexpression or H19 transcription deficiency results in fetal overgrowth (Sasaki et al., 2000). Genomic imprinting is erased and established during gametogenesis and is maintained throughout the lifetime of an organism (Simon et al., 1999; MacDonald and Mann, 2014). Thus, the restoration of a diploid genome in fertilized embryos and mutually compensatory expression of monoallelic parent-specific imprinted genes ensure normal growth and development of early embryos. However, genomic imprinting is not effectively maintained in cloned embryos, resulting in the aberrant expression of imprinted genes that gives rise to development defects such as placental hypertrophy and fetal abortion and death (Figure 2, Genome imprinting) (Mann et al., 2003; Shi et al., 2003; Yang et al., 2007; Wei et al., 2010; Zhang et al., 2014; Huan et al., 2015b). For example, hypomethylated H19/Igf2 imprinting results in increased H19 transcription and suppresses the growth of cloned fetuses, whereas Igf2 overexpression mediated by hypermethylated H19/Igf2 imprinting leads to their overgrowth. Therefore, disrupted genome imprinting during the development of cloned embryos results in developmental abnormalities and death in cloned offspring, constraining the cloning efficiency.

Female mammals have two X chromosomes, whereas only one is present in males. In order to balance gene dosage, female mammals silence one X chromosome through the activity of the Xist gene product, a long non-coding (lnc)RNA on the inactive X chromosome that recruits transcriptional repressors such as PcG proteins (Latham, 2005; Galupa and Heard, 2015). During normal embryonic development of female mammals, both X chromosomes are active and XCI occurs at the blastocyst stage, resulting in random inactivation of the X chromosome in the inner cell mass (ICM). Meanwhile, the paternal X chromosome is inactivated in the trophoblast (Yang et al., 2007; Payer, 2016). During SCNT, the inactivated X chromosome in female donor cells is reactivated during early development of cloned embryos, with XCI occurring at the blastocyst stage. In theory, XCI should occur randomly in the ICM, with the trophoblast exhibiting XCI as in the donor cells. However, irrespective of the sex of cloned embryos, DNA methylation level of Xist is lower than that in fertilized embryos, and the consequent upregulation of Xist expression represses the transcription of numerous X-linked genes (Figure 2, XCI) (Xue et al., 2002; Nolen et al., 2005; Inoue et al., 2010; Xu et al., 2013; Yuan et al., 2014; Ruan et al., 2018). Such abnormal XCI has also been detected in the placenta and carcass of dead cloned animals and could be due to the absence of a H3K27me3 mark in the Xist promoter following SCNT, and H3K9me3 may also determine the expression level of Xist in cloned embryos (Xue et al., 2002; Inoue et al., 2010; Inoue et al., 2017b; Ruan et al., 2018). Therefore, the abnormal XCI pattern seriously affects the development of cloned fetuses and placentas.

As epigenetic modification regulates gene expression, disrupted epigenetic modification during SCNT leads to the abnormal transcription of genes related to development in cloned embryos. The persistently high expression of donor somatic cell-specific genes and failure to activate genes related to embryo development are against SCNT-mediated nuclear reprogramming (Matoba et al., 2014; Liu W. et al., 2016). Therefore, epigenetic modification status determines gene expression levels and the developmental potential of cloned embryos, further suggesting that only the full and effective reconstruction of epigenetic modifications during SCNT-mediated nuclear reprogramming can support the full-term development of cloned embryos.

## Strategies for Enhancing the Development of Cloned Embryos by Improving Epigenetic Reprogramming

Presently, the aim of SCNT-mediated nuclear reprogramming-related research is to improve epigenetic reconstruction in cloned embryos, as the degree of epigenetic reprogramming determines the developmental competence of cloned embryos (Niemann, 2016).

Improving DNA methylation reprogramming has been applied in cloned embryos (Enright et al., 2003; Huan et al., 2015a; Liao et al., 2015). One way in which this is accomplished is by recapitulating the DNA methylation pattern of normal fertilized embryos using DNA-demethylating agents or by Dnmts including Dnmt1 and Dnmt3l gene silencing. The application of DNA demethylation reagents and Dnmts knockdown have successfully ameliorated genome DNA methylation and histone modification in cloned embryos. The nucleoside analog 5-aza-2′-deoxycytidine (5-aza-dC) is incorporated into the genome during DNA replication, inhibiting DNMT1 activity and resulting in DNA hypomethylation (Enright et al., 2003). Genomic DNA hypomethylation by 5-aza-dC treatment has been shown to improve the development of cloned embryos, whereas Dnmt1 or Dnmt3l knockdown in somatic cells or cloned embryos increases gene-specific DNA methylation and histone modification reprogramming and, consequently, developmental competence (Diao et al., 2013; Huan et al., 2015a; Liao et al., 2015; Song et al., 2017b). Additionally, the expression level of Tet3 in oocytes has been shown to be positively correlated with the developmental competence, and Tet3 overexpression in donor cells restores normal DNA hypermethylation and increases the full-term development of cloned embryos (Han et al., 2018). Therefore, ameliorating DNA methylation reprogramming in cloned embryos is a feasible strategy to enhance cloning efficiency.

Modifying histone marks is another approach for increasing the development competence of cloned embryos. Hdac inhibitor treatment increases histone acetylation and opens up the chromatin structure, which facilitates the binding of transcription factors that activate genes involved in early embryonic development. Hdac inhibitors have been used to improve the developmental ability of cloned embryos. For example, trichostatin A (the class I and II Hdac inhibitor), scriptaid (a synthetic Hdac inhibitor with low toxicity), and valproic acid all increase histone acetylation levels, especially H3K9ac and H3K14ac, improve gene expression levels in cloned embryos, and thus enhance SCNT-mediated nuclear reprogramming (Enright et al., 2003; Kishigami et al., 2006; Bui et al., 2011; Costa-Borges et al., 2010; Liu et al., 2012; Zhai et al., 2018). These results suggest that histone acetylation is beneficial for the development of cloned embryos. The increased H3K4me3 has been shown to improve the epigenetic modifications and the developmental efficiency of cloned embryos (Zhai et al., 2018). H3K9me3 has been reported to be a barrier for SCNT-mediated nuclear reprogramming, and removal of H3K9me3 through injection of Kdm4 mRNA activates the appropriate expression of repressed genes and increases the developmental competence of cloned embryos (Antony et al., 2013; Matoba et al., 2014; Chung et al., 2015; Liu et al., 2018; Weng et al., 2019). Importantly, the positive effect of histone acetylation on cloning efficiency could also be mediated through H3K9me3 removal (Matoba et al., 2014). Moreover, the loss of H3K9me3 can also be realized by introducing protamines in the nuclei of donor somatic cells, holding great potential to improve cloning efficiency (Iuso et al., 2015). Additionally, blocking H3K27me3 has been shown to promote nuclear reprogramming and embryonic development following SCNT (Xie et al., 2016; Zhou et al., 2019). Therefore, improvements in histone modification can correct the expression pattern of genes required for the normal development of cloned embryos and greatly enhance cloning efficiency.

Importantly, with the enhanced development of cloned embryos induced by histone modification improvements, genome imprinting in cloned embryos, fetuses, and offspring is also effectively maintained, suggesting that epigenetic modifications form mutually regulatory networks (Cervera et al., 2009; Xu et al., 2013; Huan et al., 2015b; Inoue et al., 2017a). H19 knockdown in abnormal imprinting fetal fibroblasts has also been shown to rescue damaged imprinting and the reduced development of cloned embryos (Song et al., 2017a). Therefore, restoring normal epigenetic marks and expression of imprinted-related genes can directly or indirectly ensure genome imprinting and the normal development of cloned embryos.

Targeting XCI is another strategy for improving developmental potential in SCNT. When Xist is deleted, the pattern of X-linked gene expression is corrected in cloned embryos, and the birth rate of cloned mammals is improved (Inoue et al., 2010; Matoba et al., 2011; Ruan et al., 2018). The inhibition of Xist also results in a remarkable improvement in the development of male cloned embryos (Zeng et al., 2016; Yang et al., 2019). Therefore, Xist deletion or knockdown restores X-linked gene expression patterns in cloned embryos and increases cloning efficiency.

**FIGURE 3:**
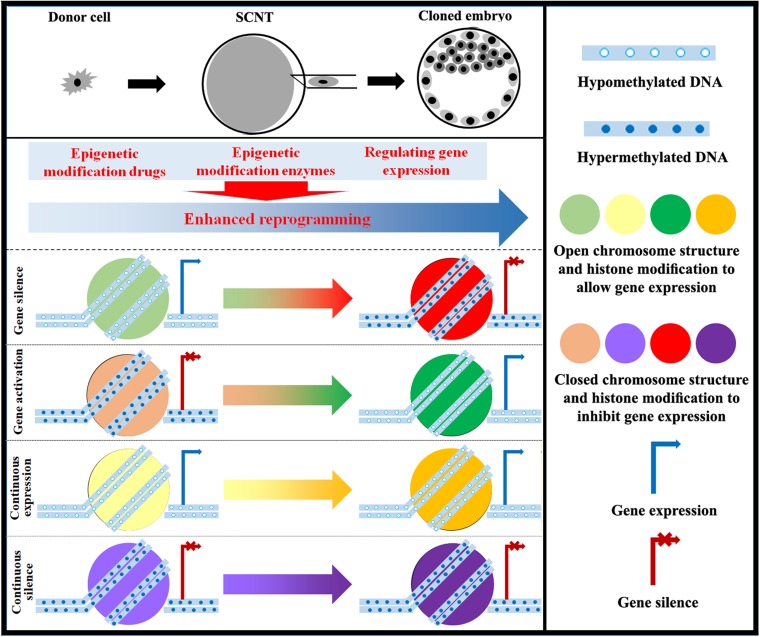
Strategy for improving epigenetic reprogramming during SCNT. The application of epigenetic modification drugs and the regulation of enzymes involved in epigenetic modification as well as associated genes can induce changes in DNA methylation, chromosome structure, histone modification, genomic imprinting, and XCI in cloned embryos close to those in normal fertilized embryos. Targeting epigenetic reprogramming to activate or silence genes can yield cloned embryos with high developmental competence.

With the understanding of epigenetic modifications during SCNT, researchers have successfully cloned macaque monkeys (Liu et al., 2018). Therefore, the existing evidence indicates that the epigenetic status of cloned embryos is an important determinant in cloning efficiency, and improving epigenetic modifications can be a good strategy to support the successful long-term development of cloned embryos (Figure 3).

## Role of lncRNAs in SCNT-Mediated Epigenetic Reprogramming

An increasing number of studies have shown that the degree of epigenetic reprogramming determines the developmental potential of cloned embryos (Niemann, 2016; Matoba and Zhang, 2018). However, the molecular regulatory network involved in SCNT-mediated epigenetic reprogramming remains unclear. Therefore, exploring the molecular mechanism underlying nuclear reprogramming induced by SCNT and clarifying how highly differentiated somatic cells effectively become pluripotent cloned embryos through the epigenetic reprogramming process are areas of great interest.

LncRNAs are gene transcripts longer than 200 nucleotides that do not encode proteins but nonetheless play a critical role in gene regulation in nearly all physiologic processes, as well as in cell fate determination during development (Pauli et al., 2011; Chen and Zhang, 2016; Wang et al., 2018). A comprehensive and systematic exploration and analysis of lncRNA function has become a frontier in the field of life science (Flynn and Chang, 2014). As mentioned above, H19 and Xist have been shown to regulate the development of cloned embryos, suggesting that lncRNAs play a key role during SCNT-mediated nuclear reprogramming (Inoue et al., 2010; Song et al., 2017a). Exploring the molecular mechanism of lncRNAs in mediating epigenetic reconstruction during SCNT can provide new ideas for improving cloning efficiency.

Recent studies have shown that lncRNAs participate in many epigenetic modification processes, such as DNA methylation, histone modification, genome imprinting and XCI, and regulate the activation or silencing of genes according to cell function requirements (Mercer and Mattick, 2013; Holoch and Moazed, 2015). During DNA methylation reprogramming, lncRNAs can interact with enzymes related to DNA methylation or demethylation (Dnmts and Tets), and determine DNA methylation reconstruction to regulate gene expression. When a gene needs to be activated, lncRNAs recruit DNA demethylation-related enzymes, such as Tets, to the gene promoter and help to achieve gene DNA demethylation, and when a gene needs to be silenced, lncRNAs interact with Dnmts to establish and maintain DNA methylation of a gene promoter (Bao et al., 2015; Hamazaki et al., 2015; Wang et al., 2015; Zhou et al., 2015; Kimura et al., 2017). Therefore, lncRNAs can interact with the DNA methylation reprogramming-related enzymes to regulate DNA methylation reprogramming and regulate gene expression. LncRNAs can also alter gene expression by regulating histone modification. Studies have demonstrated that lncRNAs recruit the TrxG proteins to catalyze H3K4me3 and enhance gene transcription or PcG proteins to silence gene expression through H3K27me3 (Tsai et al., 2010; Wang et al., 2011; Liu et al., 2015). LncRNAs are also involved in the regulation of genomic imprinting, as evidenced by the finding that every genomic imprinting center contains at least one lncRNA, such as H19 and Meg3 in the H19/Igf2 and Dlk1/Meg3 imprinting regions, respectively, that regulates monoallelic parental-specific gene expression through epigenetic silencing (Barlow and Bartolomei, 2014). During XCI, Xist has been displayed to recruit PcG protein to certain gene loci, establish H3K27me3 modification and DNA methylation, and lead to gene silencing on the X chromosome (Lee, 2009; Inoue et al., 2017b). Therefore, lncRNAs can determine epigenetic modification construction.

**FIGURE 4:**
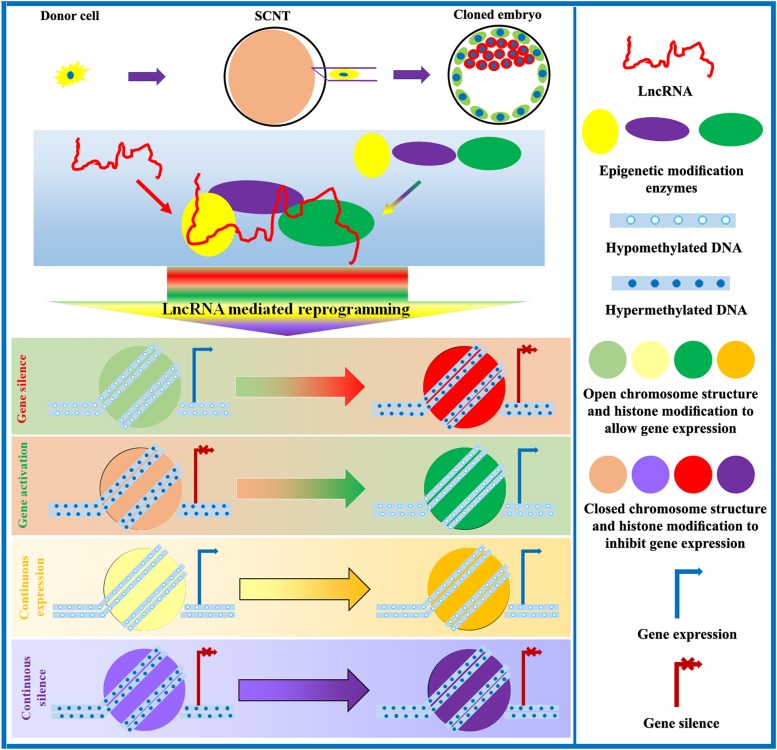
Role of lncRNAs in the regulation of SCNT-mediated epigenetic reprogramming. LncRNAs interact with epigenetic modification enzymes to modulate DNA methylation, chromosome structure, histone modification, genomic imprinting, and XCI, thereby controlling gene expression and promoting the development of cloned embryos.

After SCNT, epigenetic and gene expression profiles undergo substantial changes in cloned embryos. Naturally, lots of differentially expressed lncRNAs exist during the development of cloned embryos. However, research on key lncRNAs during the SCNT process is very limited. Encouragingly, the existing studies have suggested that lncRNAs can regulate the developmental competence of cloned embryos (Inoue et al., 2010; Song et al., 2017a; Ruan et al., 2018). Studies of parthenogenetic and semi-cloned mice have shown that these animals also express different levels of H19, which are associated with variable patterns of epigenetic modification and gene expression and influence developmental potential (Kono et al., 2004; Zhong and Li, 2017). Additionally, in iPSCs, lncRNAs are also shown to either promote or inhibit the reconstitution of epigenetic modifications during nuclear reprogramming (Kim et al., 2015). Therefore, lncRNAs could determine the developmental competence of cloned embryos. Given the importance of lncRNAs during SCNT-mediated epigenetic reprogramming, exploring and clarifying the underlying molecular mechanism of lncRNA-mediated epigenetic reprogramming during SCNT could lead to improvements in cloning efficiency (Figure 4).

## Future Research and the Application of SCNT

It is known to be difficult to produce cloned embryos, and their developmental competence remains poor. Moreover, studies on the factors that regulate epigenetic reprogramming during SCNT, especially those investigating lncRNAs, are still limited. To enhance the developmental potential of cloned embryos, a systematic analysis of the factors and mechanisms involved in SCNT is required. In recent years, with technological advancements, particularly the application of single-cell transcriptome sequencing, great progress has been made in discovering and identifying the reprogramming factors related to the development of cloned embryos. To date, lots of novel genes and lncRNAs have been revealed (Bai et al., 2016; Liu X. et al., 2016; Wu et al., 2018). However, the relevant reprogramming factors, including the emerging lncRNAs that regulate the developmental potential of cloned embryos, have not yet been deeply explored. Therefore, more detailed studies are needed to elucidate the molecular mechanisms underlying SCNT-mediated epigenetic reprogramming in order to improve cloning efficiency.

Efforts to improve cloning efficiency have also promoted the application of SCNT technology. Presently, a series of agriculturally and economically important animals can be cloned, which could not only enable the protection of endangered species but also accelerate the utilization of livestock. Gland bioreactors can be created through SCNT to produce therapeutic proteins. Animal models can also be generated through SCNT to investigate the pathogenesis of human diseases. Moreover, with the CRISPR/Cas9-mediated genome editing technology, SCNT can produce desired animals or models for specific applications. In short, if cloning efficiency is greatly improved, the application of SCNT technology will be more extensive.

## Conclusion

SCNT has important theoretical and practical research value. In this review, we present our understanding of SCNT-mediated nuclear reprogramming, especially the factors contributing to low cloning efficiency, and that incomplete epigenetic reprogramming leads to the low developmental potential of cloned embryos. We further demonstrate that the application of epigenetic modification methods can improve cloning efficiency. We also describe the regulation of epigenetic reprogramming by lncRNAs and provide a new research perspective in the field of SCNT-mediated epigenetic reprogramming. The elucidation of these mechanisms has enhanced cloning efficiency and expanded the application of SCNT technology in agriculture, regenerative medicine, and other areas. We believe that with further advances in technology, more molecular mechanisms will be revealed to enhance the development of cloned embryos, and improved cloning efficiency will promote the extensive application of SCNT technology.

## Author Contributions

YH designed the manuscript. XW, JQ, and JL wrote the manuscript. YH, HH, and ZL provided the writing guidance and revised the manuscript. All authors read and approved the final manuscript.

## Conflict of Interest

The authors declare that the research was conducted in the absence of any commercial or financial relationships that could be construed as a potential conflict of interest.
